# A narrative review of the measurement methods for biomechanical properties of plantar soft tissue in patients with diabetic foot

**DOI:** 10.3389/fendo.2024.1332032

**Published:** 2024-07-29

**Authors:** Xiong-gang Yang, Zhi Peng, Xiang Liu, Xiao-liang Liu, Sheng Lu

**Affiliations:** ^1^ Department of Orthopedics, The First People’s Hospital of Yunnan Province, The Affiliated Hospital of Kunming University of Science and Technology, Kunming, Yunnan, China; ^2^ The Key Laboratory of Digital Orthopedics of Yunnan Province, Kunming, Yunnan, China

**Keywords:** diabetic foot, biomechanical properties, plantar soft tissue, shear force, measurement method

## Abstract

This article provides an overview of the development history and advantages and disadvantages of measurement methods for soft tissue properties of the plantar foot. The measurement of soft tissue properties is essential for understanding the biomechanical characteristics and function of the foot, as well as for designing and evaluating orthotic devices and footwear. Various methods have been developed to measure the properties of plantar soft tissues, including ultrasound imaging, indentation testing, magnetic resonance elastography, and shear wave elastography. Each method has its own strengths and limitations, and choosing the most appropriate method depends on the specific research or clinical objectives. This review aims to assist researchers and clinicians in selecting the most suitable measurement method for their specific needs.

## 1 Introduction

Diabetes has become one of the most serious health threats in today’s era. It has been reported that if the current trend of diabetes prevalence continues, it is estimated that by 2050, 21–33% of the US population will suffer from diabetes (1). China has the largest diabetic population in the world, with an estimated 116 million adults affected, accounting for about 25% of global diabetes cases (2). Diabetes is associated with complications affecting multiple systems in the body, such as retinopathy, kidney disease, diabetic foot ulceration (DFU), and autonomic neuropathy. Foot-related diseases, including infections, ulcers, and gangrene, are common symptoms among hospitalized diabetes patients. Among them, DFU is one of the most common complications of diabetes, with approximately 15–25% of diabetes patients experiencing DFU in their lifetime, and 5–24% of patients requiring amputation within 6–18 months after the first DFU assessment (3–5). According to the International Diabetes Federation, globally, approximately 9.1–26.1 million diabetes patients develop DFU each year (6). DFU imposes a significant burden on society, including limb disabilities in diabetic patients and the associated substantial hospitalization and healthcare costs. In the United States, the average cost of treating a DFU patient is around $13,179, totaling up to $58 billion annually (7, 8).

DFU is primarily developed based on three key pathological factors: neuropathy, trauma with secondary infection, and peripheral arterial disease (9). Peripheral neuropathy leads to intrinsic muscle atrophy in the feet, resulting in hammer toes and the creation of “high-pressure” areas beneath the metatarsal heads. In the presence of impaired skin sensation and proprioception, decreased feedback and adaptive adjustments to pain or pressure in the feet can lead to repeated micro-trauma even during normal walking, causing the protective plantar fat pad to atrophy and dislocate, ultimately leading to foot ulceration and infection (10). Diabetes-induced neuropathy is a symmetrical polyneuropathy affecting motor, sensory, and autonomic nerve functions to varying degrees. These factors contribute to abnormal gait, altered plantar pressure and shear forces, increased risk of infection, decreased injury threshold, and reduced skin healing capacity, collectively leading to DFU development. Hyperglycemia and its related metabolic changes can cause endothelial damage, elevated blood lipids, increased platelet adhesion and activation, and, over time, the development of atherosclerosis. With the progression of diffuse tibial arterial occlusion or more proximal arterial occlusion, inadequate microcirculation in the feet to maintain skin integrity can lead to ischemic ulcers and gangrene, and reduced self-healing ability for minor tissue injuries further exacerbates soft tissue damage (9). Therefore, DFU occurs and progresses as a result of the combined effects of multiple biological and mechanical mechanisms.

The foot is the first point of contact with the ground during human locomotion and bears several times the body weight as a reactive force. The plantar soft tissues serve as the primary cushioning structure that maximally reduces the transmission of impact stress to the skeletal system (11–13). In diabetic patients, a prolonged hyperglycemic environment leads to a series of chemical reactions between reducing sugars and cellular proteins, resulting in the formation of advanced glycation end products (AGEs) (14, 15). The accumulation of AGEs is a major cause of diabetic tissue pathology and physiological changes (16, 17). The buffering capacity of the plantar soft tissues depends largely on their viscoelastic properties. Therefore, if the tissues lose their viscoelasticity due to continuous AGE accumulation, their ability to absorb impact and evenly distribute loads during weight-bearing activities will be reduced (18). Increased stiffness, decreased damping effects, and lower tissue damage thresholds in the heel pad of diabetic patients have been confirmed by numerous studies. Thus, accurate measurement of the structural and biomechanical parameters of the plantar soft tissues is crucial for DFU prevention and early risk classification (19). Although the heel region is not the highest occurrence area for DFU and other pathological conditions on the sole, the heel pad (HP) has always been a more focused site among scholars when studying the material properties of the plantar soft tissues. Possible reasons include: (1) although DFU and other pathological conditions in the heel region are not the most common, once they occur in this area, they are difficult to heal and greatly affect the patient’s mobility, resulting in high treatment costs (20); (2) the unique structure of the heel pad makes it an ideal subject for biomechanical research. Compared to the forefoot fat pad, the heel pad has a more specialized structure, comprised of differentiated adipocytes surrounded by fibro-septal compartments that form a honeycomb-like pattern (20). Additionally, the heel pad has a relatively thick and large volume, making it easier to observe and capture morphological changes during gait. The exploration of testing methods for the biomechanical properties of the plantar soft tissues has been ongoing, and significant progress has been made as advancements in available tools and measurement techniques have emerged. This paper provides a comprehensive review of the development process of testing methods for the biomechanical properties of plantar soft tissues in diabetic patients. It aims to guide the establishment of novel measurement tools for assessing the biomechanical properties of plantar soft tissues, provide references for selecting more accurate and convenient testing methods in clinical practice, and assist in the prevention and early risk classification of DFU.

## 2 Structural and biological characteristics of plantar soft tissues

**Figure 1:**
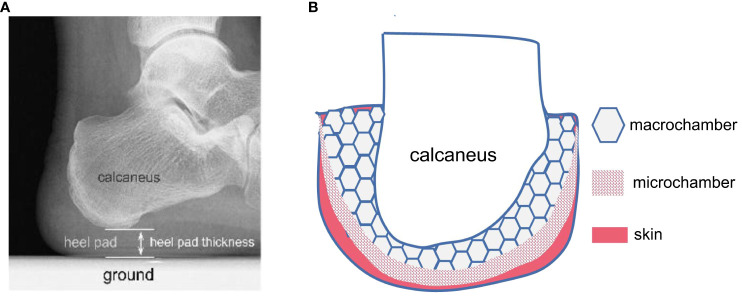
Schematic diagram of the internal structure of the heel pad (HP). **(A)** HP is a soft tissue fat pad located under the human heel, with an average thickness of 18mm. It is the first point of contact between the human body and the ground during walking. **(B)** HP exhibits a specialized honeycomb-like structure, composed of dense fibrous septa that encapsulate the fatty tissue. The main components of the fibrous septa are collagen fibers and elastic fibers, originating from the plantar fascia and terminating in the dermis, forming a completely closed cavity structure. Numerous fat cells are filled in the closed cavity formed by the fibrous septa. HP consists of an outer small compartment layer and an inner large compartment layer, exhibiting different biomechanical properties. The outer compartment layer is approximately 10 times stiffer than the inner compartment layer and undergoes minimal deformation under normal loads, while the large compartment layer is the primary structure responsible for compression deformation.

During the gait cycle, the foot plays a crucial role in force transmission as it first contacts the ground. The plantar fat pad serves as a natural “shock absorber” with energy dissipation properties, providing cushioning and damping effects. The internal structure of plantar soft tissues consists of numerous highly differentiated compartments, as illustrated in **Figure 1**. These compartments are composed of adipocytes surrounded by fibrous septa, forming closed structures that do not communicate with each other (21). Within the plantar region, from the skin layer to the bone surface, the soft tissues differentiate into shallow smaller compartments and deeper larger compartments. The smaller compartments undergo minimal deformation and exhibit approximately ten times the stiffness of the larger compartments, which mainly deform under load and significantly contribute to the viscoelastic properties of the plantar soft tissues (21).

The intact structure of plantar soft tissues is crucial for buffering external stresses imposed on them, functioning similarly to a damper that attenuates peak forces and dampens vibrations (22). During walking, a portion of the compressive or shear energy applied to the plantar soft tissues dissipates as heat, while another portion is released through elastic rebound (23). The complex differentiated structure of healthy plantar soft tissues enables them to withstand stress impacts during daily activities without sustaining damage. However, conditions such as aging, diabetes, plantar fasciitis, peripheral neuropathy, foot vascular diseases, cavus foot, rheumatoid arthritis, hormonal use, and trauma can lead to degeneration of the plantar fat pad, resulting in reduced damping effects and lowered damage thresholds. Diabetes introduces complex biochemical changes in the body, and persistent hyperglycemia and accelerated accumulation of AGEs are major factors underlying detrimental pathological changes in diabetic plantar soft tissues (24).

Morphological studies have demonstrated tissue morphological changes in the plantar fat pad of diabetic patients, including decreased volumes of adipocytes, increased thickening and fragmentation of fibrous septa, and relative decrease in fat content with increased fibrous septa content (25–30). Wang et al. (25) compared the morphological differences in the plantar fat pads beneath the first metatarsal head and heel between diabetic and non-diabetic cadaveric specimens and found that fibrous septa and dermal layers were significantly thicker in the plantar fat pads of diabetic patients, while fusion degree between dermis and epidermis and the size of adipocytes showed no significant differences. In another study by Wang et al. (26), histological and biochemical composition analyses were performed on six different regions of the plantar fat pad (big toe area, first metatarsal head area, third metatarsal head area, fifth metatarsal head area, lateral arch area, and heel area) from elderly diabetic and non-diabetic cadavers. The authors found that the most significant changes in diabetic patients, compared to the healthy population, were increased thickness of fibrous septa and increased elastic fiber content, while no significant difference was observed in fibrous septa thickness between the heel area and other regions, but elastic fiber content was markedly reduced. Waldecker and Lehr (27) conducted biopsies on the subcalcaneal fat pad tissue, but their research did not find any differences in adipocyte size between diabetic and non-diabetic individuals. Others have reported that the skin thickness (both on the foot dorsum and at other locations) is greater in diabetic patients (28, 29), and impaired gene expression related to extracellular matrix remodeling leads to decreased mechanical performance (30). Kuhns et al. (31) reported twisted and fractured collagen fiber bundles in the fibrous septa and fat cell extravasation in degenerated heel pads of elderly individuals. The most notable changes in degenerated plantar fat pads were the relative increase in the amount, thickness, and fragmentation of elastic fibers (12). These changes result in decreased damping performance, reduced energy dissipation capacity, and consequently, more energy acting on the plantar soft tissues during gait, leading to further tissue damage.

**Figure 2:**
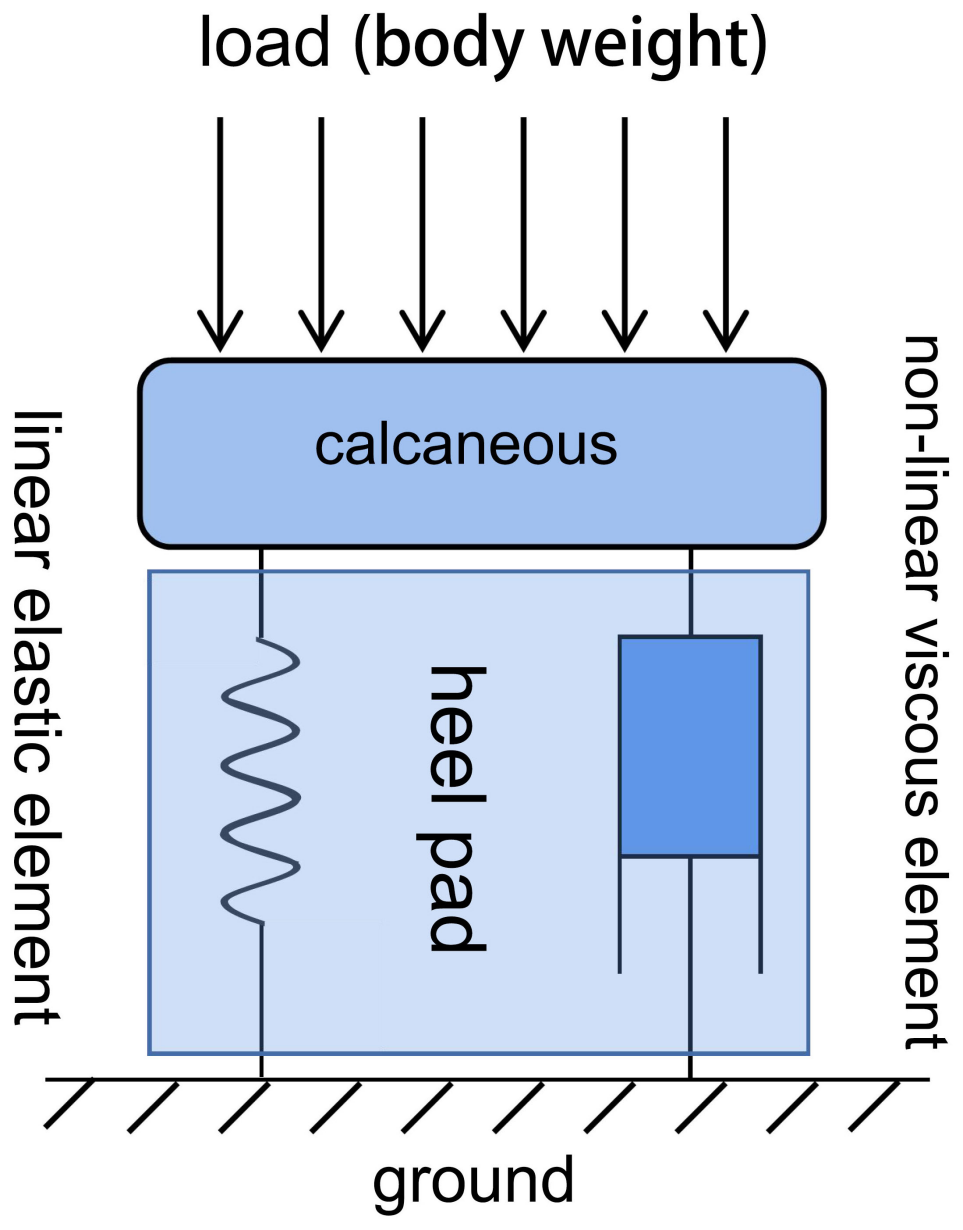
Kelvin-Voigt viscoelastic material model of the soft tissue beneath the heel, which undergoes corresponding deformation when subjected to mechanical loading. This model consists of two elements (a linear elastic element and a nonlinear viscous element) connected in parallel.

Plantar soft tissues exhibit typical viscoelastic properties and can be simplified according to the Kelvin-Voigt viscoelastic material model, composed of parallel linear elastic elements and nonlinear viscous elements (**Figure 2**). The normal damping and stress-buffering capabilities of plantar soft tissues depend largely on their viscoelasticity. During the gait cycle, the plantar soft tissues experience repeated cycles of stress loading and unloading. Due to the viscoelastic properties of the plantar soft tissues, the energy generated during impact between the foot and the ground is partially dissipated as heat during the tissues’ rebound, thereby attenuating the energy transmitted to the skeletal system. Stress-strain curves during loading and unloading cycles form characteristic hysteresis loops, with the size of the area reflecting the energy dissipation performance of plantar soft tissues. When biochemical composition and tissue morphology of plantar soft tissues change due to prolonged hyperglycemia, their viscoelastic properties are affected, further reducing their ability to absorb shock and evenly distribute loads during weight-bearing activities (18). Therefore, testing the viscoelastic properties of plantar soft tissues in diabetic foot conditions is an important approach for evaluating the progression of diabetic foot. However, existing research has primarily focused on testing the elastic properties of plantar soft tissues, often neglecting the exploration of their viscous properties. The time-dependent viscous properties of plantar soft tissues play a significant role in stress buffering and energy dissipation (32). Studies have shown that the viscous properties of plantar soft tissues are more sensitive in assessing the diabetic condition compared to other commonly used parameters such as tissue stiffness (33).

## 3 Advancements in biomechanical testing of plantar soft tissues

**Figure 3:**
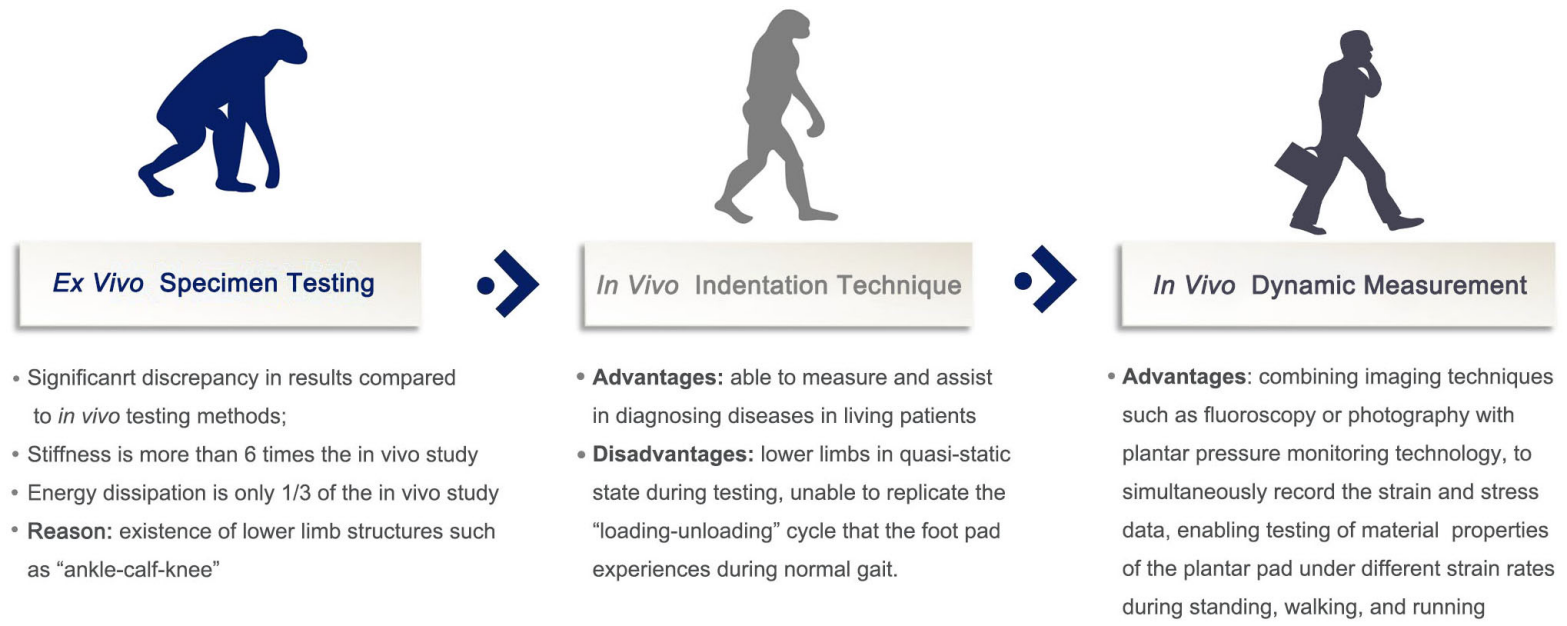
Development and pros and cons of measurement methods for soft tissue biomechanical properties of the plantar foot.

Accurate and convenient testing of the biomechanical properties of plantar soft tissues plays a crucial role in the prevention and risk stratification of DFUs. As reported by Naemi et al. (34), the mechanical properties of plantar soft tissue measured using ultrasound elastography technique can be used to improve the predictability of DFU in moderate/high risk diabetic patients. In Morrison et al. (35), the authors aimed to ascertain if B-mode ultrasound could be clinically applied to identify structural change in the diabetic foot and be utilised as an early predictor of ulceration risk. However, they found that no direct evidence was found to indicate B-mode ultrasound measures can predict soft tissue changes in the plantar foot in diabetes. With an increasing understanding of the tissue morphology and biomechanical properties of plantar soft tissues, as well as advancements in testing equipment and analysis methods, numerous methods have emerged to evaluate the material properties of plantar soft tissues. Overall, the development of these techniques has transitioned from *ex vivo* studies to *in vivo* research and from quasi-static conditions to dynamic loading. The developmental history and respective advantages and disadvantages of measurement methods for plantar soft tissue material properties are shown in **Figure 3**.

Initially, researchers primarily created standardized test specimens from excised plantar soft tissue and subjected them to compression testing using universal material testing machines to observe the compressive material properties of plantar soft tissues. Subsequently, some *in vivo* quasi-static testing methods were introduced, including ultrasound indentation, air-jet indentation based on optical coherence tomography (OCT), dynamic spherical indentation systems, and tissue ultrasound palpation systems. These methods yielded more reliable and repeatable results but were unable to replicate the dynamic loading-unloading process experienced by plantar soft tissues throughout a complete gait cycle.

In recent years, some scholars have attempted to dynamically observe the strain of plantar soft tissues during the gait cycle using X-ray imaging techniques. Concurrently, they recorded stress information using plantar pressure plates and solved for the material properties through fitting calculations. This method enabled dynamic assessment of plantar soft tissues during a gait cycle, providing results that are more representative of the true properties of the plantar fat pad. However, current research has predominantly focused on testing the vertical compressive properties of plantar soft tissues, while exploring the horizontal shear properties of these tissues still poses significant technical challenges. As a result, relevant evidence in this regard is difficult to obtain. This may be due to past neglect of shear forces and the technical difficulties associated with measuring shear force and shear strain.

### 3.1 *Ex vivo* specimen testing for biomechanical properties

Due to the limitations of early testing tools, researchers initially explored the material properties of plantar soft tissues using *ex vivo* specimens and uniaxial compression tests on universal material testing machines to measure stress relaxation and compression properties of plantar soft tissues (36–39). Alexander et al. (38) conducted initial dynamic compression testing on ex vivo specimens of foot fat pads (metatarsal pad and heel pad) from various mammals. Bennett et al. (23) performed uniaxial compression loading-unloading tests on the heel region (calcaneus + HP) of 11 cadaveric feet, observing nonlinear stiffness in all samples, with stiffness increasing as the load increased. The average stiffness measured when the load was equivalent to body weight was 1160 ± 170 kN/m, with an average compression deformation of 2.07 ± 0.29 mm. The mean energy dissipation rate (EDR) was 28.6 ± 6.9% before decalcification and 32.3 ± 5.4% after decalcification of the calcaneus. In Miller-Young et al.’s study (39), the authors prepared 8 mm diameter standard specimens of the HP from 20 cadaveric feet and subjected them to a series of unconstrained loading tests (quasi-static, 175 mm/s, 350 mm/s, and stress relaxation tests) using a material testing machine to observe the viscoelastic properties of HP and obtain constitutive equations for modeling plantar soft tissues. The results showed nonlinear viscoelastic behavior of the HP, and the experiments yielded a series of parameters suitable for finite element simulation. Ledoux et al. (36) obtained specimens of the soft tissues in six regions of the plantar surface (under the big toe, under the second metatarsal head, under the third metatarsal head, under the fifth metatarsal head, lateral arch, and heel region) from 11 cadaveric foot specimens. Compressive and stress relaxation experiments were conducted using a material testing machine on standardized 2x2 cm test specimens, and the maximum stress, elastic modulus, and EDR of the soft tissues were compared among different regions and under different strain rates. Significant differences were found in the maximum stress, elastic modulus, and EDR among the six plantar regions, with the heel region exhibiting the highest maximum stress and elastic modulus and the lowest EDR. Additionally, as the strain rate increased, the maximum stress, elastic modulus, and EDR of the plantar soft tissues also significantly increased. Pai et al. (37) used the same method to measure the force-dependent mechanical properties of the plantar soft tissues in four diabetic and age-matched non-diabetic cadaveric foot specimens. The results showed that the diabetic foot specimens exhibited higher elastic moduli than the non-diabetic foot specimens in various plantar regions and at different strain rates (average 1146.7 vs. 593.0 kPa). The material properties of the specimens demonstrated clear strain rate dependency. While *ex vivo* specimens provide convenient testing of the mechanical properties of plantar soft tissues, evidence suggests significant differences between the material properties of the heel pad measured through *ex vivo* mechanical tests compared to *in vivo* experiments, with increased stiffness and decreased EDR observed in *ex vivo* tests (23, 40–42). This difference is believed to be due to the effects of other structures of the lower limb during *in vivo* measurements (42). Therefore, *ex vivo* measurements cannot fully reflect the biomechanical properties of plantar soft tissues in physiological conditions, and *ex vivo* methods cannot provide real-time *in vivo* measurements of patients.

Furthermore, a limited number of studies have tested the shear mechanical properties of plantar soft tissues using *ex vivo* specimens (43–45). Ledoux et al. (43), Pai et al. (44), and Brady et al. (45) prepared *ex vivo* specimens of plantar soft tissues from cadaveric feet and used a material testing machine to measure the shear mechanical properties of different regions of the plantar surface. The obtained shear elastic modulus was significantly smaller than the compressive elastic modulus in the vertical direction, measuring only around 50 kPa.

### 3.2 *In vivo* testing for biomechanical properties

While the use of *ex vivo* specimens for testing the material properties of plantar soft tissues offers a straightforward and data-processing-friendly approach, it is limited to experiments conducted on cadaveric samples and cannot be performed in living subjects. As a result, direct data cannot be obtained for assessing the status of plantar soft tissues in diabetic patients and evaluating the risk of DFUs. Furthermore, *ex vivo* specimens lack the presence of other parts of the lower limb (“ankle-calf-knee”) and the internal structures of the remaining foot, leading to complete unconstrained conditions around the plantar soft tissues after detachment. These factors contribute to a significant disparity between the biomechanical properties measured in *ex vivo* testing and those observed in *in vivo* testing. This issue is commonly referred to as the “heel pad paradox” (42, 46, 47), and was initially proposed by Aerts et al. in 1995 (42). The authors conducted pendulum impact tests on live subjects and compression loading tests using universal material testing machines on *ex vivo* specimens, revealing a substantial discrepancy in stiffness (*in vivo*: 150 kN/m vs. *ex vivo*: 900 kN/m) and EDR. Additionally, Pain et al. (46) created a two-dimensional model of the lower leg and heel pad using DADS software and compared the heel pad properties with and without the involvement of the lower limb through pendulum impact tests, further confirming the existence of the “heel pad paradox” and the impact of lower limb soft tissues on heel pad property testing results.

To address this issue, *in vivo* testing of the biomechanical properties has become increasingly prevalent, with various researchers designing numerous testing methods. Most of these methods involve maintaining the subject’s foot in a quasi-static state during testing and applying different forms of loading to assess the mechanical properties of plantar soft tissues. Some researchers have also developed dynamic testing methods that involve assessing plantar soft tissues during gait cycles under X-ray fluoroscopy, allowing for the complete reproduction of the mechanical loading process experienced by the subject’s plantar soft tissues during normal walking.

In summary, attempts at *in vivo* testing of the biomechanical properties of plantar soft tissues have gained popularity, and researchers have devised various testing methods. Most of these methods involve quasi-static testing with the subject’s foot in a stationary state, applying different loading units to test the mechanical properties of plantar soft tissues. A smaller number of researchers have designed dynamic testing methods that evaluate plantar soft tissues under X-ray fluoroscopy during gait cycles, allowing for the replication of the mechanical loading experienced by the subject’s plantar soft tissues during normal walking.

#### 3.2.1 *In vivo* quasi-static measurements for mechanical properties

##### 3.2.1.1 Pendulum impact test

The pendulum impact test involves using a rod-shaped pendulum with a known mass and an accelerometer. The pendulum is suspended by a cord and swung to impact the desired area of the plantar soft tissues. The accelerometer records the acceleration data during the entire swinging process, and combined with the mass of the pendulum, it allows for the calculation of continuous displacement and force information during the impact process, which can be used to estimate the material properties of the plantar soft tissues (42, 46–48). Throughout the testing process, the subject’s foot remains fixed on the testing platform in a stationary position. Weijers et al. (47) conducted a study with 11 subjects to investigate the damping characteristics of the plantar tissues in the heel region during heel strike while controlling the venous congestion of the lower limb using a blood pressure cuff. They used the pendulum test at swinging speeds of 0.2, 0.4, and 0.6 m/s to assess the mechanical properties of the plantar soft tissues and found that the venous congestion affected the damping effect of the plantar soft tissues to some extent. As previously mentioned, Aerts et al. (42) and Pain et al. (46) also employed *in vivo* pendulum impact tests to compare the results with those obtained from *ex vivo* specimen testing in order to verify the “heel pad paradox”. In another study, Aerts et al. (48) performed pendulum impact tests on the heel regions of nine subjects and compared the mechanical characteristics between soft-soled shoes and hard-soled shoes based on the deformation and load data calculated from the pendulum mass and the negative acceleration recorded by the accelerometer. This measurement method provides a better simulation of the impact between the heel region and the ground during motion and offers advantages such as easy experimental setup, inexpensive equipment, and straightforward data processing.

##### 3.2.1.2 Dynamic spherical indentation system

*In vivo* spherical indentation tests primarily rely on stress relaxation tests to measure the viscoelastic properties of the live heel pad (HP) (19, 49, 50). Negishi et al. (19) conducted stress relaxation tests on the soft tissues of the heel region in three healthy subjects using a self-designed spherical indentation system to investigate the influence of different strain rates on the stress relaxation curves. They found significant differences in the stress relaxation curves of the plantar soft tissues under different strain rates, indicating a notable effect of strain rate on the viscoelastic properties of the plantar soft tissues. Suzuki et al. (49) determined the viscoelastic and hyperelastic material properties of the plantar soft tissues through spherical indentation tests and an analytical contact model. They obtained the stress relaxation curve of the HP through the indentation experiment and fitted the curve into the contact model of the Maxwell model to calculate the viscosity material parameter. In another study by Suzuki et al. (50), the authors derived an analytical contact model for spherical indentation tests to directly estimate the material properties of the plantar soft tissues. Through indentation experiments, they obtained the force-displacement curve of the HP. By fitting the experimental data to the stress-strain analytical solution of the spherical indentation, they successfully calculated the nonlinear material properties of the HP using the spherical indentation method.

##### 3.2.1.3 Ultrasound/MR elastography

Ultrasound elastography is another widely used approach for assessing the mechanical properties of plantar soft tissues (51–60). Based on the different elastic moduli of human soft tissues, their deformation under external compression varies. Ultrasound elastography converts the real-time changes in echo signal displacement before and after compression into color images. Tissues with lower elastic moduli appear red, indicating higher strains after compression, while tissues with higher elastic moduli appear blue, indicating lower strains. Tissues with intermediate elastic moduli appear green on the image. Naemi et al. (52) employed ultrasound shear wave elastography to test the elastic moduli of the soft tissues below the first metatarsal head, third metatarsal head, and heel region in 51 subjects with diabetes or prediabetes. They found a significant correlation between fasting blood glucose levels and plantar tissue stiffness. Lin et al. (54) utilized ultrasound elastography to examine the elastic modulus of the HP in 20 healthy subjects and 16 patients with unilateral heel pain. The results showed significantly higher elastic moduli in the affected compartmental layers (superficial, intermediate, and full layer) of the HP in patients with heel pain compared to the healthy side. Additionally, the elastic moduli of the compartments (superficial, intermediate, and full layer) were significantly elevated in heel pain patients compared to healthy subjects.

Magnetic resonance elastography (MRE) is an emerging imaging technique that can estimate the inherent elastic properties of tissues (61, 62). Information obtained from MRE can be displayed as tissue stiffness maps and provide valuable information for manual palpation. It is commonly used as a clinical tool for diagnosing breast diseases (61, 62). Weaver et al. (61) conducted *in vivo* testing of the shear modulus of the HP using MRE and found that the shear modulus of the soft tissues beneath the heel gradually increased from 8 kPa to 12 kPa as pressure was applied, while the soft tissues surrounding the heel remained around 8 kPa. The obtained shear modulus results were similar to those measured using the same method for breast adipose tissue. Cheung et al. (62) performed elastic modulus testing of the plantar soft tissues in 12 non-diabetic subjects and 4 subjects with diabetic neuropathy using MRE. The elastic modulus values for the two groups were 4.85 kPa and 5.26 kPa, respectively. MRE can detect early mechanical property changes in diabetic feet and provide a non-invasive means to monitor the progression of diabetic foot disease. Using MRE technology to monitor patients can lead to earlier detection of foot disorders, prompting proactive measures by clinicians to prevent callus formation, skin breakdown, ulcers, and eventual amputation. MRE maps can also help orthopedic surgeons and engineers identify areas with the most significant changes in plantar tissue mechanical properties and design personalized cushioning footwear/insoles to control ulcer development.

##### 3.2.1.4 Ultrasound/MR indentation technique

Ultrasound/MR indentation technique combines an ultrasound/MR probe with an indentation/compression device. The ultrasound/MR probe records the deformation and strain of the plantar soft tissues, while the loading unit on the indentation device, equipped with digital mechanical sensors, captures real-time mechanical information. By integrating these two components, the mechanical properties of the plantar soft tissues can be calculated. In recent years, numerous researchers have designed this type of measurement device and successfully applied it to test plantar soft tissues (63–73).

Chatzistergos et al. (63) designed a measurement device consisting of an ultrasound probe, a force sensor, and a manually operated ball screw loading unit to investigate the correlation between the mechanical performance of the HP in type-2 diabetes patients and clinical characteristics. They tested 35 type-2 diabetes volunteers and found a significant positive correlation between triglyceride levels and HP stiffness (r = 0.675, p<0.001), as well as a significant negative correlation between fasting blood glucose and energy absorption of the HP (r = -0.598, p = 0.002). Hsu et al. (71) developed an ultrasound transducer based on a 7.5MHz linear array and a combination of loading devices weighing 0.5→3.0→0.5kg. They conducted structural and mechanical property tests on the HP of 20 young subjects and 13 elderly subjects. The results showed that the initial thickness (2.01 ± 0.24 cm vs. 1.76 ± 0.20cm), peak strain (61.3 ± 5.5% vs. 53.3 ± 7.7%), and EDR (35.3 ± 10.0% vs. 23.7 ± 6.9%) of the HP were significantly higher in the elderly group compared to the young group. Stiffness was higher in the elderly group, although the difference was not statistically significant (p = 0.098). In another study by Hsu et al. (72), they used a linear array ultrasound transducer with a frequency range of 5–12MHz and a self-designed device capable of different loading and unloading speeds (fast loading: 10 cm/s; medium loading: 2.0 cm/s; slow loading: 2.5 cm/s) to compare the material properties beneath the metatarsal heads of the left foot between 10 middle-aged and elderly subjects (age range: 42–72) and 9 young subjects (age range: 19–35). They found that as the loading speed increased, the elastic modulus of the young subjects gradually increased from 300 kPa to around 500 kPa, while the middle-aged and elderly group did not show a significant trend, remaining around 500 kPa to 550 kPa. The EDR increased from 30% to approximately 60% in the young group and from 40% to around 70% in the middle-aged group. Overall, in most metatarsal heads, the elastic modulus of the middle-aged group was significantly higher than that of the young group. Trebbi et al. (73) established a measuring method using MR imaging and a mechanical loading plate to assess the internal compression and shear strain of soft tissues (HP, sacral soft tissues, etc.), providing guidance for risk assessment of DFUs or pressure ulcers.

##### 3.2.1.5 Other quasi-static indentation techniques

In the study by Chao et al. (74), the authors designed a jet-indentation system based on OCT and a tissue ultrasound palpation system. These two systems were used to test and compare the mechanical properties of the plantar soft tissue in the forefoot of 30 young and elderly individuals. The results revealed a strong positive correlation (r = 0.88, p<0.001) between the data obtained from the two methods. The stiffness of the first metatarsal bone was significantly higher in the elderly group compared to the young participants, while the soft tissue thickness of the first/second metatarsal bone was significantly lower in the elderly group. Kwan et al. (75) also employed a tissue ultrasound palpation system to test the soft tissue thickness and stiffness of the toes, first/third/fifth metatarsal bones, and heel region in 60 participants aged between 41 and 83 years. The results showed a significant increase in the soft tissue stiffness (p<0.001) in all five tested locations with increasing age. There was no significant correlation between age and plantar soft tissue thickness.

#### 3.2.2 *In vivo* dynamic measurement of mechanical properties

Although the methods mentioned above can achieve *in vivo* measurement of plantar soft tissue, the subjects are all in a quasi-static state, with their lower limbs fixed on the measuring device, unable to complete the gait cycle and reproduce the loading-unloading process of the plantar soft tissue during daily activity. In order to overcome this limitation, De Clercq et al. (18), Gefen et al. (76), and Wearing et al. (77) combined imaging techniques such as X-rays and plantar pressure testing plates to measure the *in vivo* biomechanical properties of the plantar soft tissue in dynamic gait. This method can monitor the strain and stress changes of the plantar soft tissue in real-time during the gait cycle and calculate the material properties of the tissue at different time phases. However, the studies by De Clercq et al. (18), Gefen et al. (76), and Wearing et al. (77) only used two-dimensional perspective photos to measure the vertical strain of the plantar soft tissue, and the accuracy of the measurement may be influenced by factors such as the shooting angle. To avoid this issue, Teng et al. (78) and Yang et al. (79) used double-plane X-ray perspective technology to continuously shoot perspective films of the plantar soft tissue from two intersecting vertical angles, and then reconstructed a three-dimensional structure of the heel in order to observe the deformation of the plantar soft tissue during the gait cycle in a three-dimensional manner. The real-time strain data was calculated, and the authors combined this with real-time stress data collected from the plantar pressure plate to fit the stress-strain data and solve for the material properties of the plantar soft tissue. This improvement in measurement equipment further improves the accuracy of the test. However, using these methods may inevitably increase additional radiation exposure risks for the subjects and experimenters, which is an important factor that researches need to consider.

### 3.3 Comparison between the described methods

When comparing the various methods for measuring the biomechanical properties of plantar soft tissue in diabetic patients, it is essential to consider their specific advantages and limitations in different scenarios. Each technique offers unique insights, but the choice depends on the research question, available resources, and the level of detail required.

#### 3.3.1 *Ex vivo* vs. *in vivo* testing

*Ex vivo* methods, such as uniaxial compression tests on universal material testing machines, are more straightforward and data-driven. However, they lack the physiological context and dynamic loading experienced *in vivo*. In contrast, *in vivo* techniques like pendulum impact tests and ultrasound elastography provide real-time data, but they may be more complex and require specialized equipment. For instance, if the focus is on understanding the impact of diabetes on tissue properties under natural loading conditions, *in vivo* methods would be more appropriate.

#### 3.3.2 Quasi-static vs. dynamic loading

Quasi-static methods like indentation testing and ultrasound elastography yield static stress-strain curves, which are useful for understanding the material properties at a specific point in time. Dynamic testing, such as X-ray fluoroscopy during gait, captures the full loading-unloading cycle, providing a more comprehensive understanding of the tissue’s behavior during functional activities. If the goal is to assess the risk of DFU development under realistic loading conditions, dynamic testing would be preferred.

#### 3.3.3 Factors influencing the choice of methods

The choice of method depends on the research objective, the need for real-time data, and the level of detail required. A combination of techniques may be necessary to obtain a comprehensive understanding of the biomechanical properties of plantar soft tissue in diabetic patients.

Additionally, socioeconomic factors, such as income levels and educational background, can influence the type of technology available to researchers and clinicians. In regions with higher levels of poverty or lower educational attainment, it may be more challenging to obtain access to advanced imaging technologies like MRI or CT, which can be expensive and require specialized training to operate. This may limit the choice of measurement methods to more traditional and affordable options, such as manual palpation or basic imaging techniques.

Access to technology can also vary between different institutions, such as academic research centers, community hospitals, and private clinics. Academic centers and larger hospitals may have more resources to invest in new technologies and equipment, while smaller or rural institutions may have limited access to these resources. This can create disparities in the quality of care and research opportunities available to patients and researchers in different regions.

Health policy and research funding play a crucial role in shaping the direction of scientific research and the implementation of new technologies in clinical practice. Changes in these areas can have a significant impact on the adoption of advanced methods for measuring the biomechanical properties of the foot, particularly in the field of diabetes-related foot complications. For instance, government policies that prioritize the prevention and management of chronic diseases like diabetes may lead to increased funding for research in this area. This could create opportunities for the development and validation of new technologies for measuring plantar soft tissue biomechanics, which could ultimately lead to improved diagnosis and treatment of diabetic foot complications. On the other hand, budget cuts or changes in research funding priorities could limit the availability of resources for new technology development and implementation. This could make it difficult for researchers to obtain the necessary equipment and expertise to conduct cutting-edge research in this area. Moreover, policies that promote interdisciplinary collaboration and knowledge translation between academia and industry could facilitate the development and commercialization of new technologies for measuring plantar soft tissue biomechanics. This could lead to faster adoption of these technologies in clinical practice and improved patient care.

## 4 Summary and future directions

Accurate and convenient testing of the biomechanical properties of the plantar soft tissue has an important role in the prevention and risk grading of DFU. With the increasing understanding of the morphology and biomechanical properties of plantar soft tissue in the academic community, as well as the continuous updates of detection equipment and analysis tools, there are various methods for evaluating the material properties of plantar soft tissue. Overall, the development of these techniques has transitioned from *in vitro* research to *in vivo* research and from quasi-static states to dynamic loading states. However, current research almost entirely focuses on testing the vertical compression properties of plantar soft tissue, and there are still significant technical difficulties in exploring the material properties of plantar soft tissue in the horizontal shear direction. Therefore, there are very few related studies reported in literature.

Secondly, at present, all the testing methods on biomechanical properties of the plantar heel pad are carried out as macroscopic biomechanical measurements of the whole structure. However, the plantar heel pad is not a completely homogeneous structure, but a specialized honeycomb structure composed of fiber elastic intervals and adipocytes, which may display differences in biomechanical properties when subjected to compression or shear stress. Currently, no study has explored the microstructures of the internal fiber intervals and adipocytes and how they affect the biomechanical properties of the heel pad when subjected to compression or shear stress. In addition, there are significant differences in the biomechanical properties between the plantar superficial layer and deep intermuscular compartment layer. The analysis of the differences in tissue structural deformation between diabetic and normal plantar soft tissues when subjected to the same level of compression or shear force has significant implications for studying the biomechanical mechanisms of DFU formation and improving DFU prevention measures. Stereology is a newly emerging interdisciplinary field that was first proposed by German biologists and mathematicians in 1961. Its core content is the quantitative study of three-dimensional structures. In recent years, with the development of modern quantitative stereology technology, its application scope has been continuously expanded, and it has now been widely used in the biomedical field. Biomedical stereology refers to the quantitative measurement of two-dimensional data obtained by measuring the geometrical information of organs, tissues, or cells using three basic geometric elements (measurable geometric information includes area, perimeter, length, width, diameter, point density, angle, etc.) and the inference of three-dimensional structural quantitative information. In recent years, some scholars have attempted to combine biomechanics with biomedical stereology and explored new methods for analyzing micro and submicroscopic biomechanical properties of soft tissues (80, 81). Eskandari et al. (80) observed the microstructural morphological changes of bovine brain white matter at different strain states under tensile and compressive mechanical loads and quantitatively analyzed the deformation of the microstructure in the tissue. Chen et al. (81) used a similar method to analyze the geometric parameters related to liver tissue injury by applying compression, tensile, and shear forces to pig liver tissue. However, there are no scholars who have applied stereological techniques to study the micro and submicroscopic biomechanics of the plantar soft tissue. Furthermore, atomic force microscope (AFM) is a more precise instrument for scanning and detecting the ultra-microscopic biomechanical properties of the sample surface, with a resolution of up to nanometer level. Therefore, future research needs to combine tissue morphological/stereological research methods with AFM and other tools to deeply explore the micro and submicroscopic biomechanical properties of the plantar soft tissue *in vitro* and compare the differences between diabetic and normal plantar soft tissues, revealing the deep impact of changes in the micro-mechanical properties on the occurrence and development of DFU.

In recent years, the integration of emerging technologies has significantly advanced the field of plantar soft tissue biomechanics research. These advancements have not only improved the accuracy and precision of measurements but also opened up new avenues for non-invasive and real-time monitoring. Wearable devices, such as smart shoes and insoles, equipped with sensors and microelectronics, can track plantar pressure distribution, gait patterns, and even detect subtle changes in tissue properties during daily activities. These devices can provide continuous, real-time data, enabling researchers to study the biomechanics of plantar soft tissue under more natural conditions. Artificial intelligence (AI) and machine learning can analyze large datasets generated by these technologies, identifying patterns and correlations that may not be apparent to the human eye. This can lead to more accurate predictions of tissue health and risk stratification for DFU. In future research, it is essential to continue exploring the potential of these emerging technologies to improve the accuracy, sensitivity, and non-invasiveness of plantar soft tissue biomechanical measurements. This includes developing novel data processing methods, integrating multiple sensing modalities, and validating these techniques in larger and more diverse patient populations. By doing so, researchers can better understand the complex interplay between diabetes and plantar soft tissue mechanics, ultimately leading to more effective prevention and management strategies for diabetic foot complications.

## Author contributions

XY: Project administration, Writing – original draft, Writing – review & editing. ZP: Writing – original draft, Supervision. XL: Writing – review & editing, Validation. XL: Writing – review & editing. SL: Project administration, Writing – review & editing.
